# A Thermoelectric Polymer Field-Effect Transistor via Iodine-Doped P3HT

**DOI:** 10.3390/mi15020172

**Published:** 2024-01-24

**Authors:** Joseph Wayne Norman, Sam-Shajing Sun

**Affiliations:** 1 Center for Materials Research, Norfolk State University, 700 Park Ave., Norfolk, VA 23504, USA; 2 Department of Chemistry, Norfolk State University, 700 Park Ave., Norfolk, VA 23504, USA

**Keywords:** field-effect transistors FET, thermoelectric, power factor PF, polymers, doping, P3HT

## Abstract

Doping can alter certain electronics, including the thermoelectric properties of an organic semiconductor. These alterations may enable viable tunable devices that could be useful in temperature sensing for autonomous controls. Here, we demonstrate a dual-modulation organic field-effect transistor (OFET) where temperature can modulate the current-voltage characteristics of the OFET and gate voltage can modulate the thermoelectric properties of the active layer in the same device. Specifically, Poly(3-hexylthiophene-2,5-diyl) (P3HT) was utilized as the host p-type semiconducting polymer, and iodine was utilized as the thermoelectric minority dopant. The finished devices were characterized with a semiconductor analyzer system with temperature controlled using two thermoelectric cooling plates. The FETs with iodine doping levels in the range of 0.25% to 0.5% mole ratio with respect to the P3HT exhibit the greatest on/off ratios. This study also observed that P3HT thin film samples with an intermediate iodine doping concentration of 0.25% mole ratio exhibit an optimal thermoelectric power factor (PF).

## 1. Introduction

Polymers appear very attractive as active layers in OFETs due to inherent advantages such as low cost [1,2] and being lightweight [3,4], flexible, [5,6], biocompatible [7], scalable [8,9], and processable at relatively low temperatures [10]. They have also been demonstrated as the active layer for a variety of applications including sensors [11,12] and displays [13,14]. Yet, when compared to other classic semiconductor devices, polymer devices are plagued by low mobility, low output current, and a small on/off ratio. Doping can greatly enhance the electrical characteristics of polymer devices and can provide further insight into charge carrier generation and transport [15].

Doping of organic semiconductors in field-effect transistors is the subject of numerous reports, including that of Wang et al., who investigated the benefits of weak dopants on a P3HT host [16]; Hu et al., who investigated n-type doping and worked within the strong electron-donating properties of the DBU species [17]; Ma et al., who doped P3HT with small quantities of F4-TCNQ and investigated the impact of charge-transfer complexes on the output and transfer characteristics of that system [18]; and Thomas et al., who looked at the impact of ions on the polymer microstructure and density-of-states functions [19]. In a similar fashion, modulation of the Seebeck coefficient with gate voltage has been investigated by Venkatenhvaran et al. with the p-type polymer semiconductor PBTTT [20]; Pernstich et al., who investigated both the temperature-dependent thermopower and carrier density-dependent thermopower in rubrene and pentacene small molecules [21]; Warwick et al., who reviewed high mobility small molecules using an on-chip architecture [22]; and by Zhang et al., who investigated the ability to predict the outcomes of chemical doping by measuring the gate voltage impact on the thermoelectric properties of a series of polymers [23]. However, temperature modulation of thermoelectric properties (Seebeck coefficient as well as power factors) and FET has not been reported via a doped organic semiconductor (OSC) active layer.

**Figure 1:**
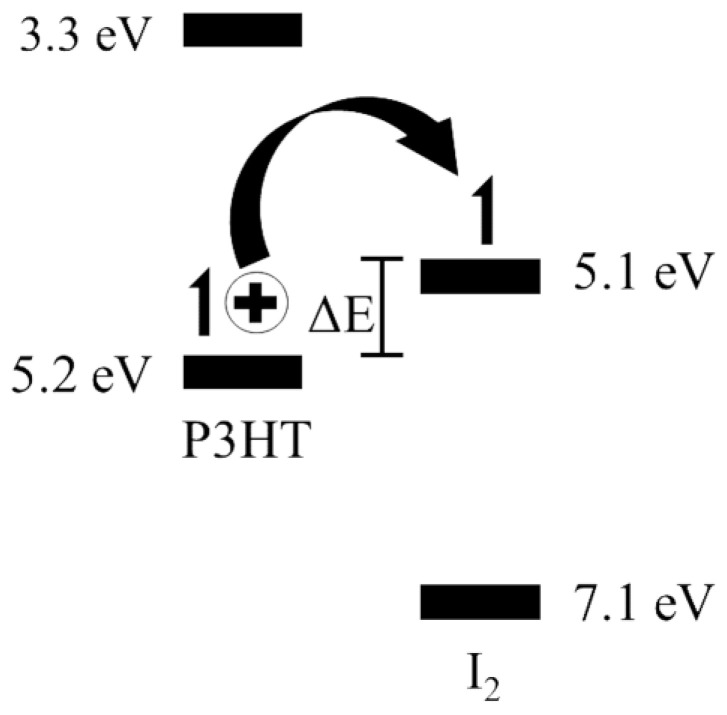
Frontier orbitals for P3HT and iodine showing the electronic transition to the dopant upon heating, which leaves a hole in the HOMO of the P3HT.

In this study, a series of pristine/undoped and iodine-doped P3HT OFETs with doping levels below 1.0% mole ratio are first evaluated in order to find the location of an optimal on/off ratio and apparent device mobility. In this binary composite system, the frontier orbitals, including the highest occupied molecular orbitals (HOMOs) and lowest unoccupied molecular orbitals (LUMOs), and energy offsets are depicted in Figure 1. Then, a series of long channel OFETs are fabricated to demonstrate a dual-modulation field-effect device whereby temperature can modulate the properties of the field-effect transistor and gate voltage can modulate the thermoelectric properties of the organic system [24,25,26,27,28]. The observed trends could aid in better understanding the role of thermoelectric dopants in charge generation and separation such as the P3HT-iodine binary system. Potential applications may include, but may not be limited to, a variety of biological systems or human wearable temperature sensors with autonomous function controls [29].

## 2. Experimental

### 2.1. Materials

Heavily-doped n-type silicon wafers were procured with a 300 nm dry thermally oxidized SiO_2_ layer and diced to a size of 15 mm × 20 mm by the fabricator. These diced wafers were utilized as the substrates for all devices. Regioregular P3HT (Reike Metals via Sigma Aldrich, St. Louis, MO, USA, SKU: 445703) and iodine (Alfa Aesar, Haverhill, MA, USA, SKU: 00158) were used for the active layer of the OFET, and octadecyltrichlorosilane (OTS) (Merck, Rahway, NJ, USA, SKU: 8.22170.0100) was used as a self-assembled monolayer to modify/improve the surface of the SiO_2_ gate dielectric. The binary composite P3HT and iodine system were dissolved in ortho-dichlolobenzene (o-DCB) and anhydrous toluene was the diluent for the OTS. All chemicals and solvents were used as provided by the manufacturers without further purification.

### 2.2. Solution and Device Fabrication

**Figure 2:**
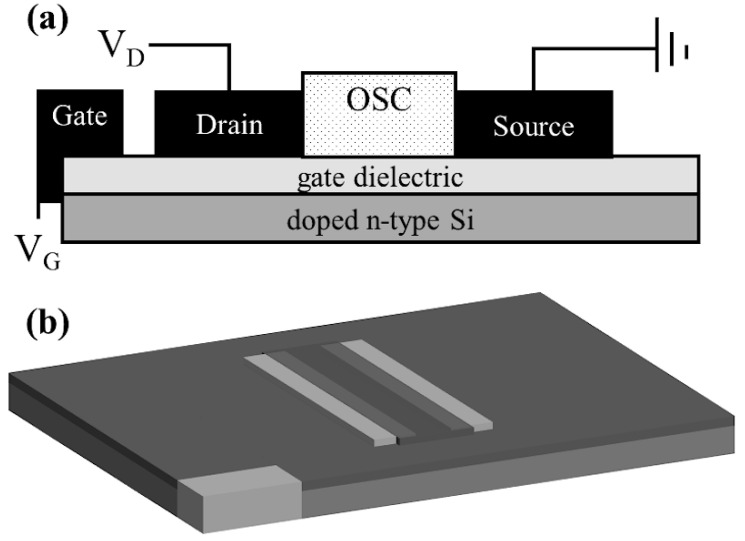
Cross-section (**a**) and orthogonal view (**b**) of a bottom-gate bottom-contact thin film transistor as investigated.

Stock solutions of P3HT in o-DCB were made at a concentration of 16 mg/mL and stock solutions of iodine in o-DCB were made at a 2% mole ratio with respect to the final blended P3HT concentration of 8 mg/mL. These stock solutions were then stirred overnight for a minimum of 8 h. After stirring, 8 mg/mL P3HT aliquots were doped with iodine via solution doping at 0.1%, 0.25%, 0.5%, and 1.0% mole ratios with an undoped P3HT aliquot also produced. These were used for an initial broad determination of an optimal doping concentration. The second doping series presented herein utilized the same solution procedure as above, but only undoped, 0.25%, and 0.5% mole ratio solutions were produced and were used for dual-modulation devices. The diced Si/SiO_2_ substrates were cleaned via immersion in an ultrasonic bath first with acetone and then with isopropanol for 30 min each before drying with a dry nitrogen stream. Source, drain, and gate contacts were thermally evaporated through a shadow mask by first depositing a 2 nm Cr adhesion promotion layer at a rate of 0.5 Å/s followed by a 50 nm Au layer at an average rate of 1.5 Å/s. For the broader doping concentration study (undoped—1%), devices have a channel length of 60 µm and a channel width of 1 mm. For the dual-modulation study, one device was produced on each substrate with a channel length of 0.580 mm and a channel width of 12 mm. After evaporation, devices were exposed to oxygen plasma for 30 s. SiO_2_ gate dielectric surfaces were then treated with OTS in anhydrous toluene via immersion for 30 min followed by a cyclohexane rinse and then dried in a dry nitrogen stream. Thin film active layers were deposited via spin coating at 1500 rpm for 120 s before being dried overnight in a vacuum oven at 80 °C to produce active layers with thicknesses of 26 nm on average. Finished devices were of the bottom-gate bottom-contact (BGBC) coplanar configuration, as shown in Figure 2a,b, where the bottom n-type Si electrode is inducted to the top surface for easy access.

### 2.3. Field Effect and Seebeck Coefficient Characterizations

Characterization of the active layers’ thermoelectric properties was performed with a Keithley 4200-SCS semiconductor analyzer (Tektronix Inc., Beaverton, OR, USA) and a Signatone 1160 probe station in atmosphere. Au probe tips were used to contact the Au source and drain contacts for voltage measurement. A temperature gradient across the OFET channel was achieved through the use of two TETech plate coolers, each connected to a TE-720 thermoelectric temperature controller (TE Technology, Traverse City, MI, USA). The temperature across the channel was verified with a FLIR A300 series IR imaging camera (Teledyne FLIR Inc., Middletown, CT, USA) and by contact. Samples were stabilized at the planned temperature gradient for five minutes prior to any data acquisition.

### 2.4. Atomic Force Microscopy

The surface morphology of the active layers was characterized using a Bruker Veeco Dimension XT atomic force microscope (Bruker Corporation, Billerica, MA, USA) set to tapping mode in air. A 5 µm × 5 µm scanning area was used in the channel of each of the devices and samples were first evaluated following fabrication and prior to any electrical characterization. The morphology was again evaluated following Seebeck, output, and transfer characterization in a location as close to the original scan area as possible. All height images were processed using the same methodology and exported so maximum heights were presented on the same scale. Roughness values were calculated from flattened images after processing.

## 3. Results and Discussion

### 3.1. Optimization of Doping Concentration Range

**Figure 3:**
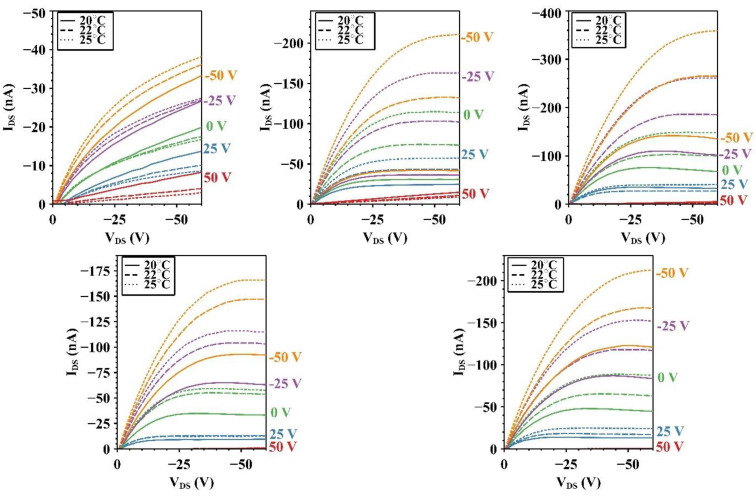
Output characteristics of undoped (**top left**), 0.1% I_2_ (**top center**), 0.25% I_2_ (**top right**), 0.5% I_2_ (**bottom left**), and 1% I_2_ (**bottom right**) devices from at 20 °C (solid line), 22 °C (dashed lines), and 25 °C (dotted lines).

Output curves for the undoped device and the four doping concentrations (0.25, 0.1, 0.5, and 1%) investigated as part of the optimization study are presented in Figure 3 for temperatures of 20 °C, 22 °C, and 25 °C. For a more direct comparison of the output tests, each of the five graphs is presented on a nanoampere scale. From quick visual analysis, it can be seen that both the 0.1% and 0.25% devices exhibit great increases in output current at elevated temperatures of 22 °C and 25 °C. For this particular set, a significant gate leakage was present and noted for the majority of doped device gate voltage levels; however, devices were still comparable as the degree of gate leakage across devices was comparable.

**Figure 4:**
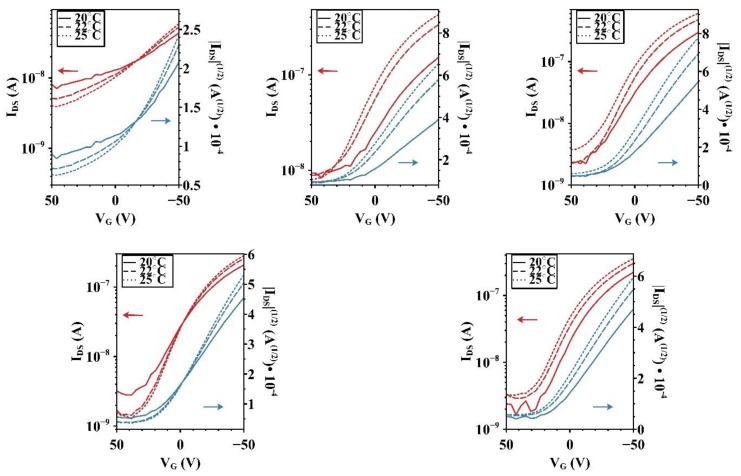
Transfer curves of undoped (**top left**), 0.1% I_2_ (**top center**), 0.25% I_2_ (**top right**), 0.5% I_2_ (**bottom left**), and 1% I_2_ (**bottom right**) devices from set 95 at 20 °C, 22 °C, and 25 °C taken in the saturation region at V_DS_ = −50 V.

Saturated mobility for each of the five devices at V_DS_ = −50 V was derived, and the off voltages can also be viewed in the transfer plots of Figure 4.

**Figure 5:**
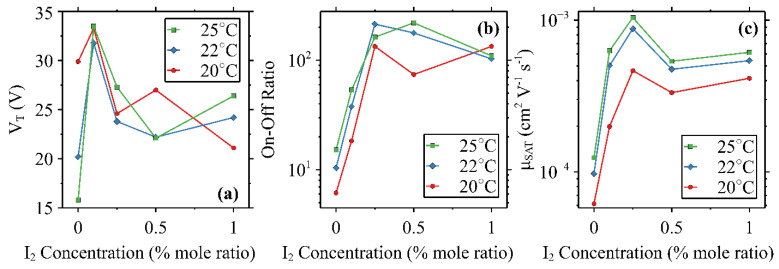
Threshold voltage (V_T_) (**a**), on/off ratio (**b**), and saturated mobility (µ_SAT_) (**c**) with respect to iodine doping concentration at 20 °C (•), 22 °C (◆), and 25 °C (■) in devices with a channel length of 50 µm.

The studies at iodine dopant concentrations below 1.0% mole ratio were performed in order to further characterize optimal parameters in both field-effect modulation and thermoelectric property modulation within that range in the in-plain XY direction FET device geometry. Thermoelectric properties extracted from the transfer curves taken in the saturation region (V_DS_ = −60 V) at 20 °C, 22 °C, and 25 °C are presented in Figure 5. The 0.1% device exhibited the greatest threshold voltage (V_T_) at all temperatures and is displayed in Figure 5a. As can be seen from the on/off ratios in Figure 5b, a maximum of 2.14 × 10^2^ is observed for the 0.25% device at 22 °C and a maximum of 2.20 × 10^2^ is observed for the 0.25% device at 25 °C. These values are within the expected range of comparable P3HT-based devices, which can vary over several orders of magnitude depending on the concentration [30] or gate dielectric [31]. Saturated device mobility values are presented in Figure 5c, and, similarly, exhibit maximums for each of the three tested temperatures for the 0.25% sample. The apparent device mobility (also called FET mobility) increases with temperature in both undoped and iodine-doped devices, as expected for temperature-dependent mobility behavior as well as thermoelectric doping-generated charges [32,33]. A slight dip at all temperatures for the 0.5% samples suggests further investigation may be necessary to further understand the impact of iodine doping on the mobility of the P3HT and iodine binary system. Previous work with a P3HT, iodine, and PCBM ternary dual-functional photoelectric-thermoelectric system revealed a peak conversion efficiency in samples doped with 5% mole ratio iodine [34], but the primary direction of charge transport differs between those reported solar cell devices (z-axis) and the OFETs reported here (xy plane). The general trend downward at 1% for both on/off ratio and mobility suggests further focus of this study should be directed toward doping concentrations of 0.25% or 0.5%.

### 3.2. Temperature Modulation of the Dual-Modulation OFET

**Figure 6:**
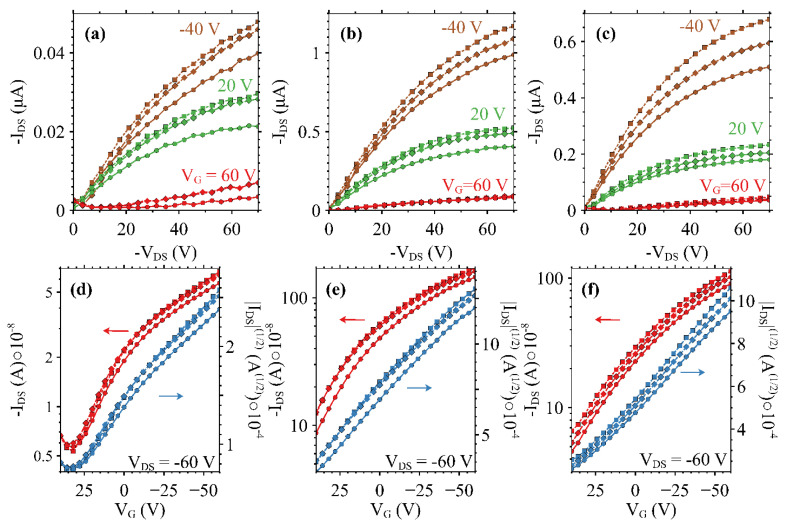
Output and saturated transfer curves for undoped (**a**,**d**), 0.25% (**b**,**e**), and 0.5% (**c**,**f**) mole ratio iodine-doped P3HT OFETs at 20 °C (•), 22 °C (◆), and 25 °C (■), where left red color arrow and data correspond to left axis of source-drain currents, and right blue color arrow and data correspond to right axis of charge mobility.

Output and transfer characteristics in saturation (V_DS_ = −60 V) for devices on which dual modulation is exhibited are displayed in Figure 6. Output curves for three uniform surface temperatures (20 °C, 22 °C, and 25 °C) are presented in Figure 6a–c, while transfer curves at the same three temperatures are presented in Figure 6d–f. An increase in conductivity is expected as the temperature is increased due to the incoherent or hopping type charge transport, which is dominant in disordered soft materials [35,36]. The addition of iodine in the doped samples acts as an electron trap as the temperature is increased, which provides additional free holes available to move through the HOMO of the P3HT. Both iodine-doped devices outperform their undoped counterpart by at least a factor of 10 over the same range of gate voltages. The 0.25% iodine-doped device exhibited the greatest output current of the three devices, reaching into the microampere range in saturation at 22 °C, and 25 °C at a V_G_ = −40 V. Transfer curves of the doped devices exhibit a well-defined linear slope from which the values of Table 1 were calculated. A maximum on/off ratio of 3.91 × 10^1^ is observed for the 0.25% device at 25 °C, and the on/off ratios of both doped devices exceeded those of the undoped device at all tested temperatures. The presented on/off ratios may be underestimated due to a truncated range over which the test was executed out of an abundance of caution to prevent damage during characterization.

### 3.3. Gate Voltage Modulation of the Thermoelectric Properties

**Figure 7:**
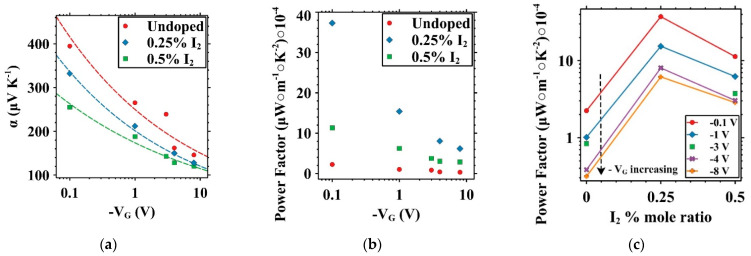
Seebeck coefficient vs. V_G_ trendlines for undoped (red circle •), 0.25% (blue diamond ◆), and 0.5% (green square ■) mole ratio iodine-doped P3HT OFETs (**a**), power factor vs. V_G_ (**b**), and power factor vs. iodine doping levels of different Vg (**c**).

Modulation of the thermoelectric properties for each of the three devices with channel lengths of 580 µm is presented in Figure 7a–c. Each point presented in Figure 7a represents a slope calculated from a series of three measurements at three different temperature gradients created across the source and drain contacts of the OFET according to the equation α=−ΔV/ΔT. As gate voltage is increased (to the negative for p-type transport), a decrease is observed in the Seebeck coefficient for both undoped and doped devices, as reflected in Equation (1). Likewise, as doping concentration is increased, a decrease in the Seebeck coefficient is observed for devices tested at the same gate voltage. Thermoelectric power factor vs. gate voltage is presented in Figure 7b and is calculated following the equation $PF=\alpha^{2}\sigma$ using values interpolated from the range of gate voltages over which the output curves were acquired at 20 °C. A maximum value of 3.7 × 10^−3^ µW·m^−1^·K^−2^ at −0.1 V was observed in the 0.25% iodine-doped device, exhibiting the greatest power factor across the tested voltage range. The 0.5% sample had the second highest values, followed by the undoped device. Figure 7c additionally presents the thermoelectric power factor with respect to the iodine doping levels of each of the three devices. Figure 7b,c shows the significance of the doping-induced conductivity gains on the thermoelectric power factor and performance of these OFETs compared to that of the undoped device. We posit that the primary driver for the increase in conductivity of the system upon heating is due to the increased number of charge carriers with both an increase in doping concentration and an increase in gate voltage. Per the Seebeck coefficient α, as expressed in Equation (1), the Seebeck coefficient in the system is inversely proportional to the charge carriers’ densities (*N*). Therefore, while the undoped sample may have a higher Seebeck coefficient among the samples tested, the 0.25%, and to a lesser degree, the 0.5% iodine-doped sample, have greater thermoelectric power factors (PFs), which are proportional to the square of Seebeck and the electrical conductivity, as reflected in Equation (2). It is expected that a maximum power factor (PF) may be identified at some gate voltage less than those presented here and could be cause for further work due to the trade-off between the Seebeck coefficient’s inverse proportionality to the charge density [31].

(1) $$\alpha =\left(8\pi^{\frac{8}{3}}k_{B}^{2}m^{*}T\right)/\left(3^{\frac{5}{3}}h^{2}qN^{\frac{2}{3}}\right)$$

PF = α^2^σ (2)

### 3.4. Atomic Force Microscopy

**Figure 8:**
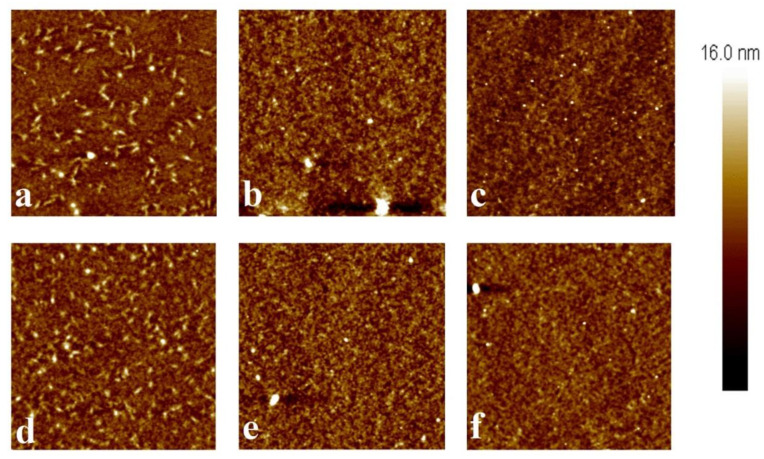
AFM height images for undoped (**a**,**d**), 0.25% (**b**,**e**), and 0.5% (**c**,**f**) both before (top row) and after (bottom row) electrical characterization and thermal cycling.

**Figure 9:**
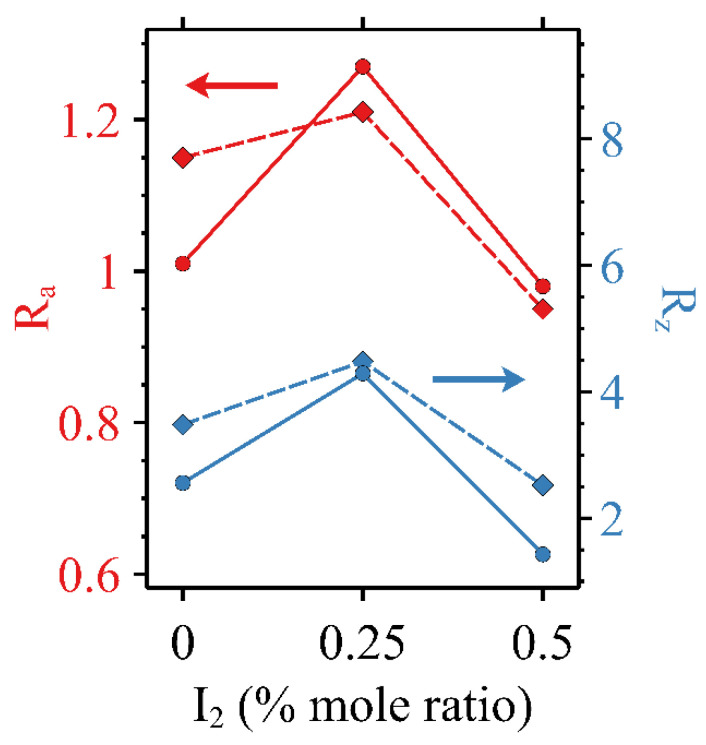
R_a_ (y_1_ axis, top red color curves) and R_z_ (y_2_ axis, bottom blue color curves) values vs. iodine dopant concentration calculated from AFM height data before (•) and after (◆) electrical characterization and thermal cycling.

To understand the impacts of the high voltage from electrical characterization and heating on the microstructure, AFM imagery was captured before and after Seebeck, output, and transfer curves were acquired. AFM images are shown in Figure 8, where the top row (Figure 8a–c) was taken before any other characterization and the bottom row (Figure 8d–f) was taken after Seebeck, output, and transfer tests were performed. No significant changes were observed in the undoped (Figure 8a,d), 0.25% (Figure 6b,e), or 0.5% (Figure 8c,f) iodine-doped. That is, the major visual characteristics of either of the sets of images did not exhibit net increases or decreases in either the size or quantity of the features observed. It should be noted that a direct comparison is difficult to achieve given the scan size of 25 µm^2^. A quantitative analysis was performed on the basis of surface roughness, however. Figure 9 displays the R_a_ and R_z_ values for the OFETs, where the 0.25% mole ratio iodine-doped specimen exhibits a greater surface roughness average and range than either of the other two active layers. It has been proposed that this may be due to an increase in the degree of halogen bonding present at low concentrations of iodine [28].

## 4. Conclusions

Pristine and iodine-doped P3HT OFETs were studied at various doping levels, temperatures, and gate voltages, which modulated the field-effect and thermoelectric characteristics, respectively. Doping P3HT with small quantities of iodine around 0.25% mole ratio improved the on/off ratio over that of pristine/undoped devices by an order of magnitude and improved the conductivity of all devices compared to that of the pristine/undoped device. AFM images revealed that there were no obvious or visible changes in the microstructures of any samples due to electrical characterization or changes in temperature; thus, the conductivity changes of P3HT OFET can be attributed mainly to charge carrier concentration. The thermoelectric Seebeck coefficient decreases with respect to both an increase in iodine doping levels and gate voltage due to an increase in the charge carrier concentrations in the active layer, as expected. However, a maximum power factor (PF) was observed at an intermediate 0.25% iodine doping, which can be attributed to an increase in electrical conductivity contribution and a decrease in Seebeck coefficient contribution toward the PF.

## Figures and Tables

**Table 1:** Threshold voltage (V_T_), saturated mobility (µ_SAT_), and on/off ratio for undoped, 0.25%, and 0.5% iodine-doped P3HT dual-modulation OFETs.

Doping (% Mole Ratio)	Sample Surface T (°C)	V_T_ (V)	µ_SAT_ (cm^2^V^−1^s^−1^)	On/Off Ratio
Undoped	20	57.6	5.13 × 10^−5^	1.01 × 10^1^
Undoped	22	56.3	6.51 × 10^−5^	1.09 × 10^1^
Undoped	25	55.3	6.40 × 10^−5^	1.25 × 10^1^
0.25	20	68.0	9.28 × 10^−4^	1.66 × 10^1^
0.25	22	76.3	9.14 × 10^−4^	3.38 × 10^1^
0.25	25	74.3	1.01 × 10^−3^	3.91 × 10^1^
0.5	20	58.2	5.91 × 10^−4^	3.70 × 10^1^
0.5	22	59.3	6.51 × 10^−4^	3.73 × 10^1^
0.5	25	60.5	7.04 × 10^−4^	3.61 × 10^1^

## Data Availability

Additional data, including electronic copies of the author’s earlier related publications and/or student dissertations, may be available in certain online databases or may be obtained directly from the author or from the library of the author’s organization where the student thesis/dissertation hard copies may be housed.
